# Evaluation of Beeswax Supplementation on Productive Performance of Growing Assaf Lambs

**DOI:** 10.3390/vetsci10090574

**Published:** 2023-09-15

**Authors:** Hamed Mohamed Gaafar, Matteo Dell’Anno, Luciana Rossi, Mohamed Kamel Mohsen, El-Sayed Mohamed Abdel-Raouf, Mostafa Mohamed El-Nahrawy, Abdeen Wajeeh Amer

**Affiliations:** 1 Animal Production Research Institute, Agricultural Research Center, Dokki, Giza P.O. Box 33717, Egypt; hamedgaafar@gmail.com (H.M.G.); mostafaelnahrawy6@gmail.com (M.M.E.-N.); 2 Department of Veterinary Medicine and Animal Sciences–DIVAS, University of Milan, Via dell’Università 6, 26900 Lodi, Italy; matteo.dellanno@unimi.it; 3 Department of Animal Production, Faculty of Agriculture, Kafrelsheikh University, Kafr El-Sheikh P.O. Box 33516, Egypt; mohamed.mohsen@gmail.com (M.K.M.); elsayedabdelraouf138@gmail.com (E.-S.M.A.-R.); 4 Animal Production Specialist at the National Campaign to Advance Camel Productivity Project, Desert Research Center, Cairo P.O. Box 11756, Egypt; abdeenamer@gmail.com

**Keywords:** assaf lambs, beeswax, digestibility, rumen fermentation, serum metabolism, growth performance, economic efficiency

## Abstract

**Simple Summary:**

Beeswax is a by-product of the bee honey industry obtained from the honeycomb of the honeybee and other bees as the crystalline substance formed from honey sugars. From the harvesting of alfalfa, a large quantity of beeswax is commonly available in Egypt. This study evaluated the effects of the supplementation of beeswax on performance, nutrient digestibility, rumen fermentation, blood metabolites, and sustainability in terms of feed costs in Assaf lambs. Eighteen lambs were allotted to three experimental groups fed with a basal diet supplemented with 0, 2, and 4 g of beeswax/day for 90 days. The results revealed an increased performance in the beeswax-supplemented groups, resulting in a higher efficiency and lower feed cost and thus optimizing farming efficiency and profitability. In addition, beeswax inclusion in the feed formulation enhanced the nutrient digestibility by enhancing rumen fermentation and decreasing the ammonia emissions. In conclusion, the use of 4 g/day of beeswax supplementation in growing Assaf lambs could promote zootechnical performance, nutrient digestibility, rumen fermentation and thus lower the cost of feed formulation and support the sustainability of lamb farming.

**Abstract:**

The aim of this work was to assess the effects of beeswax supplementation on growth rate, feed intake, nutrient digestion, rumen fermentation, blood parameters, and economic sustainability in Assaf lambs. Eighteen growing Assaf (5 months old) lambs were separated into three experimental groups (*n* = 6 lambs/group). The lambs were fed a basal diet without supplementation (G1) or supplemented with 2 and 4 g beeswax/head/day in G2 and G3 groups, respectively. Zootechnical performance was evaluated over a 90 day period. Feed digestibility was assessed in faeces through the acid insoluble-ash method, and rumen liquor was collected to measure ammonia (NH_3_-N) and total volatile fatty acid (TVFA) levels. Blood samples were obtained for the titration serum metabolites by colorimetric tests. The findings showed that G3 had an improved performance compared to the other groups (*p* < 0.01). The lambs in G3 revealed the highest nutrient digestibility and feed use, followed by G2, and G1. G3 recorded the highest economic efficiency followed by G2 and G1 (*p* < 0.01). The TVFA, acetate, and propionate concentrations were higher and the pH values, NH_3_-N, and butyrate concentrations were lower in G3 compared to G2 and particularly to G1 (*p* < 0.01). The concentrations of total protein, globulin, and glucose were significantly higher with 4 g beeswax (*p* < 0.05). However, albumin, cholesterol, total lipids, urea, creatinine, glutamic oxaloacetic transaminase (GOT), and glutamate pyruvate transaminase (GPT) concentrations as well as the albumin to globulin ratio decreased significantly with both levels of beeswax (*p* < 0.05). The addition of beeswax at the level of 4 g/head/day for growing Assaf lambs significantly improved the growth performance, digestibility, rumen fermentation, and blood serum parameters in addition to the economic efficiency.

## 1. Introduction

For centuries, bee-derived products such as honey, propolis, and beeswax have been used as natural therapeutics in folk medicine healing due to their properties and their high content of bioactive compounds [1]. Today, there is renewed interest in apitherapy due to its preventing and healing properties for wounds, rheumatism, and gastrointestinal disorders [2]. Worker bees produce beeswax through abdominal glands, and their production commonly peaks during late spring to create honeycombs [3]. Beeswax is the substance that forms honeycomb structures; bees secrete wax to build honeycombs for honey storage. It is a by-product of the bee honey industry that is obtained after the honey has been filtered, and large quantities are available in Egypt after harvesting alfalfa. Higher thicknesses and masses were achieved with alfalfa honey, due to the moderate weather during the period from 25 May to 10 June and the big flowering area of Alfalfa [4]. Hydrocarbons, free fatty acids and alcohols, palmitic-derived hydroxymonoesters, 15-hydroxypalmitic and oleic acids, linear wax monoesters, and complex wax esters comprising 15-hydroxypalmitic acids and diols are some of the chemical components that vary by bee species and geographic region [5,6]. These include hydrocarbons (12–16%) with a primarily long chain of C27–C33, and above all heptacosane, nonacosane, hentriacontane, pentacosane, and tricosane, which make up the majority of beeswax [7]. There are also fatty acids that are free (12–14%) and have a C24–C32 chain length. Linear wax monoesters and hydroxymonoesters generated predominantly from myeloid, 15-hydroxypalmitic, and oleic acids have been found to consist of 35% to 45% hydroxymonoesters and linear wax monoesters, with chain lengths ranging from C40 to C48 [8]. 

Beeswax has been employed as an additive for several industries in products, or during processing. In pharmaceutics, it has been exploited as a thickener, binder, and carrier. Beeswax products have been used since ancient times both for their nutritional value and for a broad spectrum of therapeutic purposes. Beeswax products are deemed to be a potential source of natural antioxidants which can counteract the effects of oxidative stress underlying the pathogenesis of many diseases [1,9].

The livestock system plays a key role in food security, and the Agriculture Organization of the United Nations (FAO) reported that animal production supplies 34% of the total protein intake globally [10]. It has been estimated that future population growth will lead to a transition towards an increased need for products of animal origin, and livestock production is expected to grow 14% by 2029 [11]. The future challenge will thus be to sustain this increased need, which will require a higher surface of agricultural soil for feed production, which could increase the competition between food and feed production. It is thus fundamental for future sustainability, in line with the One Health principles, to produce more and more efficiently whilst using fewer resources. According to the sustainable development goals listed in 2030 Agenda, the circular economy is crucial for achieving this [12]. Nutritional ecology strategies are thus necessary for ensuring the health of humans and animals, and also for supporting a sustainable production [13,14].

The role of diet has a great impact on the quality of life, and the use of functional products with high concentrations of bioactive molecules of a natural origin is preferable to synthetic products [15,16]. Animal nutrition, through its functional role, is aimed at increasing performance and also supporting health status, thus decreasing the occurrence of pathologies and antibiotic use in livestock [15]. In ruminant nutrition, beeswax is an interesting by-product as a natural source of bioactive compounds which can impact positively on animal health. Beeswax is utilized as an ingredient in numerous products and industrial operations, including the food, cosmetics, and candle sectors, and it has significant biological activity [17]. In the field of pharmacology, many recent studies have been published discussing the benefits of honeycombs in treating caries and toothaches, as well as their antibacterial qualities [9]. In nutrition, beeswax can be considered interesting for its nutritional benefits and healing properties [18]. Moreover, since beeswax modifies rumen bacterial fermentation, improves digestibility, and increases growth performance in cattle and poultry [17], it could be beneficial to ruminants as nutritional supplements.

The aim of this study was to determine the effects of beeswax supplementation on growth performance, feed intake, digestibility, rumen fermentation, blood metabolites, and economic efficiency in growing Assaf lambs.

## 2. Materials and Methods

### 2.1. Animals, Beeswax, and Experimental Diets

The experiments were performed according to the guidelines of a local ethics committee for animal care and welfare (Number 08/2016 EC). Eighteen growing male Assaf lambs (sheep breed specialized in meat production resulting from the crossbreeding of Awassi with East German Friesian) with an average age of 5 ± 0.04 months and a live body weight of 23.47 ± 0.27 kg were allotted to three groups of six animals each for a total duration of 90 days. A beekeeper in Saint Catherine, in the South Sinai Governorate of Egypt, provided the beeswax for this investigation. The beeswax additive (E901, Regulation EC 1333/2008) used in the following trial was mainly composed of 57.4% total esters (40.8% monoesters, 9.2% hydroxymonoesters, 7.4% diesters), 15.7% total hydrocarbons (12.8% alkanes, 2.9% alkenes), 18% total free fatty acids, and 0.6% total free fatty alcohols [19]. The wax was extracted and frozen for easier grinding and then re-mixed with wheat bran in a 2:1 ratio to ensure a homogeneous distribution and to prevent any clump formation.

Lambs were housed in an open yard with a shed area of about one third of the total area and individually fed (at 8 a.m.) a total mixed diet comprising 50% concentrate feed mixture (CFM), 30% corn silage (CS), and 20% alfalfa hay (AH) to meet the recommended requirements for growing sheep [20]. Lambs in group 1 were fed the basal ration without any wax supplementation (control group; G1), whereas lambs in group 2 (G2) and group 3 (G3) were supplemented with 2 and 4 g of beeswax/head/day, respectively. The CFM consisted of 34% wheat bran, 31% yellow maize grain, 28% undecorticated cottonseed meal, 4% molasses, 2% limestone, 1% sodium chloride, and 0.3% mineral and vitamin premix. Table 1 shows the proximate composition of the feed ingredients and basal diet used for the study.

A total mixed diet was offered every day at 8 a.m. Water was freely available throughout the day. Every two weeks, the amount of total mixed diet was adjusted to account for variations in live body weight.

### 2.2. Zootechnical Performance Evaluation

After a 16 h fast, lambs were weighed biweekly before feeding in the morning. Dietary conversions were calculated as kg of DM (dry matter), TDN (total digestible nutrients), CP (crude protein), and DCP (digestible crude protein) per kg of live body weight (LBW). The ratio between the income from daily weight gain and the cost of daily feed consumed was used to assess the economic efficiency. The cost in 2020 for CFM was LE 5000 per ton, LE 600 per ton for CS, LE 3500 per ton for AH, LE 65 per kg for beeswax, and LE 55 per kg for live body weight gain.

### 2.3. Feed Digestibility

Three digestion trials were undertaken for three experimental groups to determine the nutrient digestion and feeding values of the tested diets using acid insoluble ash (AIA) as a natural marker [21]. Fecal samples were taken from the rectum of each animal twice a day with 12 h interval for a total of 5 days. Feed and fecal samples were analyzed according to the AOAC methods [22] for the content of dry matter (DM), crude protein (CP), crude fiber (CF), ether extract (EE), and nitrogen free extract (NFE). Neutral detergent fiber (NDF), acid detergent fiber (ADF), and acid detergent lignin (ADL) were further determined in feeds according to Van Soest et al. [23]. Nutrient digestibility was calculated using the equations of Schneider and Flat [24].

$$\mathrm{DM}\;\mathrm{digestibility}\;\%=100-(100\times \frac{\mathrm{AIA}\%\;\mathrm{in}\;\mathrm{feed}}{\mathrm{AIA}\%\;\mathrm{in}\;\mathrm{feces}})$$

$$\mathrm{Nutrient}\;\mathrm{digestibility}\;\%=100-(100\times \frac{\mathrm{AIA}\%\;\mathrm{in}\;\mathrm{feed}}{\mathrm{AIA}\%\;\mathrm{in}\;\mathrm{feces}})\times (\frac{\mathrm{Nutrient}\%\;\mathrm{in}\;\mathrm{feces}}{\mathrm{Nutrient}\%\;\mathrm{in}\;\mathrm{feed}})$$

Total digestible nutrients (TDN) and digestible crude protein (DCP) were calculated by standard formula of McDonald et al. [25]. 

TDN = (CP × CPD + CF × CFD + EE × EED × 2.25 + NFE × NFED)/100

DCP = (CP × CPD)/100

CPD: Crude protein digestibility, CFD: crude fiber digestibility, EED: ether extract digestibility, NFED: nitrogen free extract digestibility.

### 2.4. Rumen Liquor Collection and Evaluation

Rumen liquor samples were collected from three lambs each in group 3 h after feeding using a rubber ruminal probe. Rumen liquor was strained through double layers of cheese cloth and immediately measured for pH using an Orian digital pH meter. Ammonia nitrogen (NH_3_-N) was determined according to the AOAC method [23]. Total volatile fatty acids (TVFAs) were determined according to the Warner method [26] and VFA fractions were determined in rumen liquor according to Filípek and Dvořák [27].

### 2.5. Blood Metabolites Profile

Blood samples were collected after 90 days of the feeding trial from the jugular vein of each lamb by vacuum tubes without anticoagulants three hours after the morning feeding and left to clot at room temperature. The blood samples were centrifuged at 1500 rpm for 10 min to obtain the serum. The blood serum content of total protein, albumin, globulin (by difference), glucose, cholesterol, total lipids, urea, creatinine, glutamic oxaloacetic transaminase (GOT) and glutamate pyruvate transaminase (GTP) were actually determined calorimetrically by spectrophotometer (Milton Roy company spectronic 20 D) using commercial diagnostic kits (test-Combination-Pasteur lap, Rockland company, Philadelphia, PA, USA).

### 2.6. Statistical Analysis

The data gathered were statistically analyzed using IBM SPSS Statistics 28.0 (IBM Corp, Armonk, NY, USA) [28]. A General Linear Model was customized to guide users through one-way ANOVA. Duncan’s tests inside the SPSS program were used to look for significant variations in the mean values of dietary treatments at the significance level of *p* ≤ 0.05.

## 3. Results

### 3.1. Growth Performance, Feed Intake, Feed Conversion, and Economic Efficiency

The results of growth performance are reported in Table 2. The findings revealed that the initial body weight was comparable for all the groups considered. On the other hand, G3 recorded the highest final body weight, average daily gain and total weight gain compared to all experimental groups (*p* < 0.01). G2 showed a significantly lower performance compared to G3, but increased growth compared to G1 (*p* < 0.01). The average daily gain of G2 and G3 increased by 16.75% and 35.81% compared to G1, respectively.

Feed conversion efficiency by lambs improved significantly (*p* < 0.05; Table 2) in the beeswax-supplemented group, showing lower FCR within raising the inclusion level. Lambs in G3 revealed the lowest DM, TDN, CP, and DCP values required per one kg live weight gain followed by G2, but G1 revealed the highest amounts (*p* < 0.01).

The economic efficiency of lambs (Table 2) showed that G3 recorded the highest feed cost and the lowest cost of gain followed by G2, and G1 (*p* < 0.01). On the other hand, G3 showed the highest price for weight gain (*p* < 0.01), net revenue, and economic efficiency followed by G2, but G1 registered the lowest values. Beeswax supplementation in G2 and G3 increased the net revenue by 25.06% and 54.10% compared to G1, respectively.

The total intake and consumption of CFM, CS, AH, DM, and CP, were significantly higher in G3 compared to G1, and G2 did not differ from G1 and G3 (*p* < 0.05). The intake of TDN and DCP was the highest in G3 followed by G2, and the lowest values were registered in G1 (*p* < 0.01) (Table 3).

### 3.2. Nutrients Digestibility

Nutrient digestibility was the highest in G3 for all considered parameters and feeding values, followed by G2, and then G1 which presented the lowest values (Table 4; *p* < 0.01). 

### 3.3. Rumen Liquor Parameters

**Figure 1:**
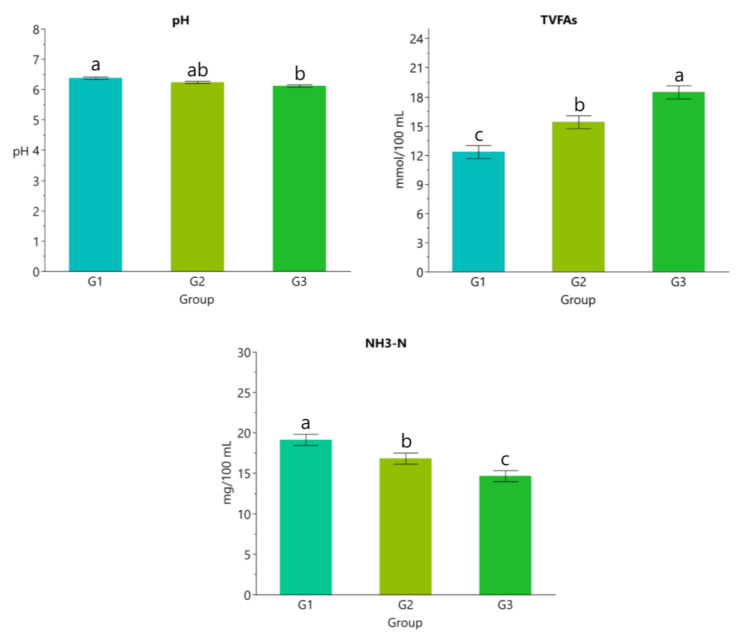
Rumen liquor pH value, total volatile fatty acids (TVFAs), and ammonia nitrogen (NH_3_-N). Data are presented as means ± standard error of the means (SEM). ^a,b,c^ Different lowercase letters indicate statistically significant differences (*p* < 0.05).

**Figure 2:**
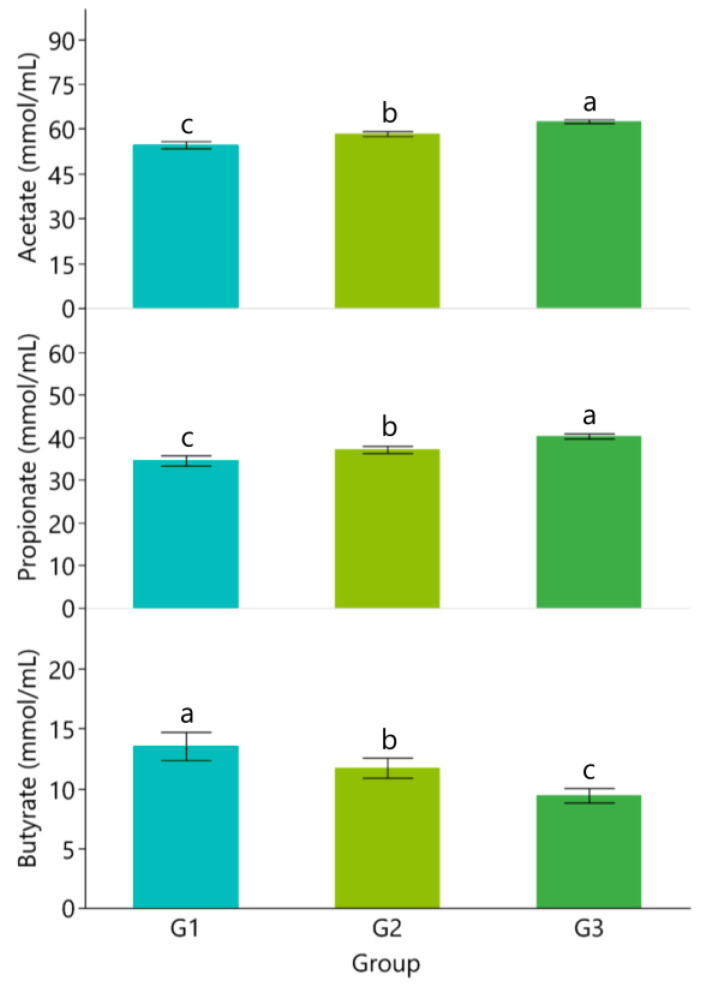
Concentrations of acetate, propionate, and butyrate in rumen liquor of Assaf lambs. ^a,b,c^ Different lowercase letters indicate statistically significant differences (*p* < 0.01).

The results in Figure 1 show that concentration of TVFA, acetate, and propionate increased with beeswax supplementation levels while pH and concentrations of NH_3_-N and butyrate decreased (*p* < 0.05; Figure 1 and Figure 2).

### 3.4. Blood Serum Parameters

Blood serum parameters are presented in Table 5. The concentrations of total protein, globulin, and glucose were significantly higher in the serum of G3 compared to G1 at *p* < 0.05. However, albumin, cholesterol, total lipids, urea, creatinine, GOT, and GPT concentrations, as well as the albumin to globulin ratio, were the lowest in G3 followed by G2; meanwhile, the highest values were registered in G1 (*p* < 0.05).

## 4. Discussion

This study evaluated the use of beeswax supplementation in feed on performance, nutrient digestibility, rumen fermentation, blood metabolites, and economic sustainability in growing Assaf lambs.

Growth performance revealed that the G3 group showed the highest performance in terms of final body weight, total gain, and ADG. In contrast, the G1 group showed the lowest performance compared to the other groups. Although the detailed composition of the beeswax used in the present study was not analyzed, the results found might be attributed, at least partly, to the presence of bioactive compounds such as remains of pollen, metamorphosis wastes, and other compounds with antimicrobial, antioxidant, and other protective effects that possibly can promote animal health and performance [7]. Indeed, the antimicrobial activity of beeswax has been extensively documented in European and Asian holistic remedies for centuries, having been shown to be effective against *Staphylococcus aureus*, *Salmonella enterica*, *Candida albicans*, and *Aspergillus niger* [29,30].

Additionally, recent studies have found that isorhamnetin derivates, kaempferol derivates, myricetin derivates, quercetin derivates, chlorogenic acid, and tiliroside obtained from beeswax byproducts exert antimicrobial and radical scavenging activity [31] and, thus, might contribute to the observed improved animal performance with beeswax supplementation. The antioxidant effect could also have contributed to the registered enhancement in animal performance [32]. In particular, a significant increase in body weight and feed conversion ratio was observed in Nellore bulls supplemented with propolis extract (containing 0.054 mg/g of total flavonoids) compared to a monensin-supplemented diet [33]. Redoy et al. [34] observed a higher live weight gain from 18% to 26% using a flavonoid-rich herbal supplementation compared to a control diet group. The average daily gain of growing lambs significantly increased twofold with polyphenol supplementation in the flavonoid-rich grape pomace group compared to the control group [35]. The content of total flavonoids of beeswax was reported at levels of 0.37 µg/g (1.9% 3,4-di-O-caffeoylquinic acid, 6.0% kaempferol, 3.4% protocatechuic acid-O-hexoside, 85.7% pinobanksin, and 3.0% apigenin) [36] and earlier studies have revealed the significant positive effects of flavonoid-rich extracts on weight gain [37]. A detailed characterization of the beeswax used in the present study would be useful to better acknowledge the effects observed.

Feed conversion efficiency showed the same trend as growth performance, revealing lower values for DM, TDN, CP and DCP per kg of body weight by increasing the beeswax supplementation. The feed conversion ratio is important for meat production when evaluating the feed conversion efficiency, and smaller feed conversion ratio values indicate a higher efficiency of feed use. Beeswax additive improved growth performance and reduced the amount of feed required for each kg weight gain. We have identified a paucity of information on the contribution of key biological processes, including appetite regulation, post-ruminal nutrient absorption, and cellular energetics and metabolism to the efficiency of feed utilization. Increasing the amount of feed consumed after the supplementation of beeswax suggests an improvement in the palatability of the feed, an increase in the animal’s appetite, or a higher growth rate. Also, our study showed that beeswax supplementation at 4 g/head/day reduced the cost of gain as well as increasing the output of gain and all economical parameters. In addition, a previous study obtained an increased return and economic efficiency with bee bread extract supplementation (2.5 and 5 mL/animal/day) in Zaraibi goats considering the price of milk yield [38].

The ingestion rate of DM and CP showed higher values in G3 compared to G1, and G2 revealed comparable values to G3 and G1; however, TDN and DCP were different among experimental groups. Similarly, DM intake by lactating goats was higher in a 3% beeswax-supplemented diet followed by 1% beeswax-supplemented diet compared to the control [39]. This effect on feed intake could be attributed to the antimicrobial molecules that have been reported to stimulate the feed efficiency, digestibility, and growth performance through the modulation of the rumen microbial environment [40]. In addition, the nutrient digestibility was significantly lower for the analyzed parameters in G1 compared to the beeswax-supplemented groups. The apparent digestibility of forage DM is considered an important index for evaluating the use of fiber sources in ruminants [38]. The results observed might be due to the components of beeswax which stimulate the activity of rumen microorganisms. In line with our study, the supplementation of propolis extracts in late pregnant ewes enhanced the nutrient digestibility, thereby, modulating rumen fermentation and microbial protein synthesis [41].

The rumen liquor analysis revealed a modulation of pH as well as TVFAs and NH_3_-N concentrations, thus further supporting a possible effect on ruminal microflora. In rumen liquor samples of beeswax-supplemented groups compared to the control lambs, TVFAs increased significantly, while NH_3_-N and pH decreased. Rumen fermentation is a complex process involving animal physiology and microbiota [15]. Ruminants establish a symbiosis between host and microbial community, thus ensuring the supply of protein, vitamins, and fatty acids to satisfy the nutritional requirements. The production of energy and protein digested in the abomasum mostly originate from ruminal fermentations [42]. It was reported that up to 50% of the host energy requirements and 90% of proteins are provided by microbial cells in normal conditions for ruminant animals [43], thus the efficiency of fermentations should be considered pivotal for animal health and performance in polygastric species. Few studies have evaluated the effect of honey by-products on rumen fermentation; however, ethanolic extract of propolis has been used in vitro, showing a preventive effect on nitrogen excretion and ammonia emission by increasing N utilization in the rumen [44]. A total of 70% of propolis extract increased the production of butyric acid and decreased the ammonia concentration by modulating the ratio bacteria/protozoa in the ruminal environment. The study of Ozturk et al. [45] suggests that bioactive compounds from propolis, partially present in beeswax, could be applied in ruminant feeding to reduce ammonia in rumen and emission into the environment. These findings are in line with higher digestibility, suggesting that beeswax supplementation could modulate rumen fermentations, possibly interacting with microorganisms and influencing feed utilization.

In our study, blood serum metabolites showed a modulation after the supplementation of beeswax for 90 days of the experimental trial. The levels of total protein, globulin, and glucose were significantly higher and the albumin:globulin ratio was lower in G3 compared to the G1 group. In contrast, cholesterol, total lipids, urea, creatinine, GOT, and GTP values were lower in G3 compared to G1. Increased concentrations of total protein, albumin, and their ratio indicate an enhanced metabolism in response to higher feed consumption and nutrient digestibility, particularly regarding the protein component of the diet and an increased microbial protein supply from the rumen [17]. Glucose levels are mainly influenced by hepatic glycogenolysis, since ruminal fermentation ensures that only small concentrations of glucose from feed carbohydrates are consumed by the microbiota [46]. Higher levels of serum glucose are probably associated with the improved performance of beeswax-supplemented groups. Several studies have demonstrated that phenolic components from Brazilian propolis have a protective effect against oxidative stressors on hepatocytes [47,48]. El-Kholi et al. [49] observed a significant decrease in serum urea, triglycerides, cholesterol, creatinine, GOT, and GPT after beeswax and propolis supplementation in diabetic rats. In addition, the levels of GOT and GPT were lower when beeswax was added to a basal diet in goats [50]. In line with the observed decrease, a significantly increased concentration of total protein and globulin, and a simultaneous decrease in triglycerides, glutamate oxaloacetate transaminase, and glutamate pyruvate transaminase were observed with supplementation of ethanolic extract of red propolis in ewes (3 g/animal/day) during the flashing period [51]. Hansem et al. [52] found a significant decrease in cholesterol and triglycerides and increased glucose, high-density lipoprotein, and total antioxidant capacity levels of serum after propolis supplementation.

## 5. Conclusions

Dietary supplementation of beeswax at 2 and 4 g/head/day to growing Assaf lambs increased growth performance by increasing the feed intake and digestibility of nutrients and modulated rumen fermentation and serum metabolites, thus resulting in a higher economic efficiency. Our results highlight the use of 4 g/head/day of beeswax as functional feed additive for growing Assaf Lambs aimed at enhancing animal performance, efficiency, and farming sustainability. Further studies will be necessary to fully characterize the beeswax and understand the role and interaction of different bioactive compounds contained in this valuable honeybee’s byproduct.

## Figures and Tables

**Table 1:** Chemical analysis of feed ingredients and basal diet.

Item	DM%	Composition on DM%								
		OM	CP	CF	EE	NFE	Ash	NDF	ADF	ADL
Concentrate feed mixture	90.15	92.65	15.10	11.10	2.95	66.45	7.36	37.50	28.60	3.80
Corn silage	29.35	93.17	8.05	25.05	2.31	57.76	6.83	46.40	26.90	3.10
Alfalfa hay	89.86	87.90	15.32	29.28	2.70	40.60	12.10	44.30	32.70	10.20

DM: dry matter; OM: organic matter; CP: crude protein; CF: crude fiber; EE: ether extract; NFE: nitrogen free extract, NDF: neutral detergent fiber, ADF: acid detergent fiber, ADL: acid detergent lignin.

**Table 2:** Growth performance, feed utilization and economic evaluation for tested groups.

Item	Tested Groups			SEM	*p*-Value
	G1	G2	G3		
Growth performance					
Initial body weight (kg)	23.10	23.45	23.85	0.27	0.568
Final body weight (kg)	37.73 ^c^	40.53 ^b^	43.71 ^a^	0.83	0.006
Total weight gain (kg)	14.63 ^c^	17.08 ^b^	19.86 ^a^	0.67	0.005
Average daily gain (g)	162.50 ^c^	189.72 ^b^	220.69 ^a^	7.48	0.005
ADG improvement (%)	100.00 ^c^	116.75 ^b^	135.81 ^a^	4.66	0.005
Feed conversion ratio					
DM (kg/kg gain)	6.55 ^a^	5.90 ^b^	5.36 ^c^	0.15	0.001
TDN (kg/kg gain)	4.06 ^a^	3.80 ^b^	3.58 ^c^	0.07	0.002
CP (g/kg gain)	853.80 ^a^	769.13 ^b^	698.54 ^c^	20.12	0.001
DCP (g/kg gain)	547.07 ^a^	515.74 ^b^	484.15 ^c^	8.90	0.003
Economic evaluation					
Daily feed cost (LE/day)	4.43 ^c^	4.79 ^b^	5.19 ^a^	0.10	0.002
Cost of gain (LE/kg)	27.29 ^a^	25.27 ^b^	23.51 ^c^	0.51	0.001
Price of weight gain (LE/day)	8.94 ^c^	10.43 ^b^	12.14 ^a^	0.41	0.001
Net revenue (LE/day)	4.51 ^c^	5.64 ^b^	6.95 ^a^	0.32	0.001
Economic efficiency ^1^	2.02 ^c^	2.18 ^b^	2.34 ^a^	0.04	0.001
Economic efficiency ^2^	101.81 ^c^	117.75 ^b^	133.91 ^a^	4.34	0.001

^a,b,c^ Values in the same row with different lowercase letters differ significantly at *p* < 0.05; ADG improvement % = ADG of G2 or G3 × 100/ADG of G1; Prices in Egyptian pound (LE) during 2020 were LE 5000/ton concentrate feed mixture, LE 600/ton corn silage, LE 3500/ton alfalfa hay, LE 65/kg beeswax, and LE 55/kg weight gain. ^1^ Economic efficiency = price of weight gain/feed cost. ^2^ Economic efficiency = net revenue × 100/feed cost. ADG: average daily gain; DM: dry matter; TDN: total digestible nutrients; CP: crude protein; DCP: digestible crude protein.

**Table 3:** Average daily feed intake by lambs of tested groups.

Item	Tested Groups			SEM	*p*-Value
	G1	G2	G3		
As fed basis (g/head/day)					
Concentrate feed mixture	590.37 ^b^	620.94 ^ab^	655.76 ^a^	10.07	0.010
Corn silage	1088.01 ^b^	1144.36 ^ab^	1208.53 ^a^	18.56	0.009
Alfalfa hay	236.91 ^b^	249.18 ^ab^	263.15 ^a^	4.04	0.011
Total	1915.29 ^b^	2014.48 ^ab^	2127.45 ^a^	32.68	0.008
On DM basis (g/head/day)					
DM	1064.44 ^b^	1119.56 ^ab^	1182.34 ^a^	18.16	0.010
TDN	659.87 ^c^	720.83 ^b^	788.95 ^a^	17.55	0.001
CP	138.70 ^b^	145.88 ^ab^	154.06 ^a^	2.37	0.010
DCP	88.87 ^c^	97.83 ^b^	106.73 ^a^	2.38	0.001

^a,b,c^ Values in the same row with different lowercase letters differ significantly at *p* < 0.05. DM: dry matter; TDN: total digestible nutrients; CP: crude protein; DCP: digestible crude protein.

**Table 4:** Nutrients digestion and nutritional values of tested diets.

Item	Tested Groups			SEM	*p*-Value
	G1	G2	G3		
Digestion coefficients (%)					
DM	62.83 ^c^	66.14 ^b^	67.35 ^a^	0.57	0.002
OM	64.55 ^c^	67.11 ^b^	69.57 ^a^	0.63	0.002
CP	64.08 ^c^	67.05 ^b^	69.29 ^a^	0.67	0.001
CF	62.73 ^c^	66.00 ^b^	68.02 ^a^	0.66	0.003
EE	79.49 ^c^	81.19 ^b^	83.37 ^a^	0.50	0.004
NFE	64.56 ^c^	66.83 ^b^	69.49 ^a^	0.66	0.002
Feeding values (%)					
TDN	61.98 ^c^	64.39 ^b^	66.72 ^a^	0.59	0.003
DCP	8.35 ^c^	8.74 ^b^	9.03 ^a^	0.09	0.004

^a,b,c^ Values in the same row with different lowercase letters differ significantly at *p* < 0.05. DM: dry matter; OM: organic matter; CP: crude protein; CF: crude fiber; EE: ether extract; NFE: nitrogen free extract; TDN: total digestible nutrients; DCP: digestible crude protein.

**Table 5:** Blood serum metabolites for tested groups.

Item	Tested Groups			SEM	*p*-Value
	G1	G2	G3		
Total protein (g/dL)	6.63 ^b^	6,80 ^ab^	6.93 ^a^	0.05	0.034
Albumin (g/dL)	3.37 ^a^	3.20 ^ab^	3.07 ^b^	0.06	0.038
Globulin (g/dL)	3.27 ^b^	3.60 ^ab^	3.87 ^a^	0.11	0.032
Albumin: globulin ratio	1.03 ^a^	0.89 ^ab^	0.80 ^b^	0.04	0.044
Glucose (mg/dL)	63.00 ^b^	67.00 ^ab^	71.33 ^a^	1.56	0.043
Cholesterol (mg/dL)	148.33 ^a^	90.33 ^b^	50.00 ^c^	14.57	0.009
Total lipids (mg/dL)	447.33 ^a^	409.33 ^b^	372.00 ^c^	11.62	0.012
Urea (mg/dL)	68.67 ^a^	51.33 ^b^	35.67 ^c^	5.08	0.008
Creatinine (mg/dL)	1.13 ^a^	1.00 ^ab^	0.90 ^b^	0.05	0.036
GOT (U/L)	170.67 ^a^	148.33 ^b^	126.33 ^c^	6.61	0.005
GPT (U/L)	27.33 ^a^	24.00 ^ab^	20.67 ^b^	1.11	0.014

^a,b,c^ Values in the same row with different uppercase letters differ significantly at *p* < 0.05. GOT: glutamic oxaloacetic transaminase; GPT: glutamate pyruvate transaminase.

## Data Availability

All data are available within the manuscript.
